# A probabilistic pathway score (PROPS) for classification with applications to inflammatory bowel disease

**DOI:** 10.1093/bioinformatics/btx651

**Published:** 2017-10-18

**Authors:** Lichy Han, Mateusz Maciejewski, Christoph Brockel, William Gordon, Scott B Snapper, Joshua R Korzenik, Lovisa Afzelius, Russ B Altman

**Affiliations:** 1 Biomedical Informatics Training Program, Stanford University, Stanford, CA, USA; 2 Inflammation & Immunology, Pfizer Inc., Cambridge, MA, USA; 3 Hill’s Pet Nutrition, Topeka, KS, USA; 4 Division of Gastroenterology, Hepatology and Nutrition, Boston Children’s Hospital, Harvard Medical School, Boston, MA, USA; 5 Division of Gastroenterology, Brigham and Women’s Hospital, Harvard Medical School, Boston, MA, USA; 6 Department of Gastroenterology, Hepatology and Endoscopy, Brigham and Women’s Hospital, Harvard Medical School, Boston, MA, USA; 7 Department of Genetics, Stanford University, Stanford, CA, USA; 8 Department of Bioengineering, Stanford University, Stanford, CA, USA

## Abstract

**Summary:**

Gene-based supervised machine learning classification models have been widely used to differentiate disease states, predict disease progression and determine effective treatment options. However, many of these classifiers are sensitive to noise and frequently do not replicate in external validation sets. For complex, heterogeneous diseases, these classifiers are further limited by being unable to capture varying combinations of genes that lead to the same phenotype. Pathway-based classification can overcome these challenges by using robust, aggregate features to represent biological mechanisms. In this work, we developed a novel pathway-based approach, PRObabilistic Pathway Score, which uses genes to calculate individualized pathway scores for classification. Unlike previous individualized pathway-based classification methods that use gene sets, we incorporate gene interactions using probabilistic graphical models to more accurately represent the underlying biology and achieve better performance. We apply our method to differentiate two similar complex diseases, ulcerative colitis (UC) and Crohn’s disease (CD), which are the two main types of inflammatory bowel disease (IBD). Using five IBD datasets, we compare our method against four gene-based and four alternative pathway-based classifiers in distinguishing CD from UC. We demonstrate superior classification performance and provide biological insight into the top pathways separating CD from UC.

**Availability and Implementation:**

PROPS is available as a R package, which can be downloaded at http://simtk.org/home/props or on Bioconductor.

**Supplementary information:**

Supplementary data are available at *Bioinformatics* online.

## 1 Introduction

Advancements in statistical modeling combined with the ease of obtaining and generating gene expression data have led to multiple approaches to build regression and classification models to aid in diagnosis, prognosis, disease prediction, patient stratification and treatment selection (Alizadeh *et al.*, 2000; Kourou *et al.*, 2015; Tan and Gilbert, 2003). For classification, the most common approaches entail using a subset of genes to derive a signature for the phenotypes of interest (Dorman *et al.*, 2016; Huang *et al.*, 2007; Ramaswamy *et al.*, 2003). However, these gene signatures have been challenging to reproduce, particularly in heterogeneous diseases such as cancer and when there is a lack of adequate validation data (Koscielny, 2010). Additionally, many limitations arise when focusing on differentially expressed genes to construct gene-based classifiers, such as noise, measurement errors and the large number of gene hypotheses, all of which can hinder reproducibility (Novak *et al.*, 2002; Swain *et al.*, 2002). Furthermore, in complex, heterogeneous diseases such as inflammatory bowel disease (IBD), there likely exist multiple combinations of gene perturbations that result in similar phenotypes. Using pathway-based methods may overcome these challenges, as combining genes to produce pathway-based feature scores has been shown to be more robust (Guo *et al.*, 2005), and can result in fewer features, which can reduce overfitting and improve generalizability while maintaining biological interpretability.

IBD is a complex and chronic inflammatory condition of the gastrointestinal tract, which affects over 1 in 200 people in the USA (Ananthakrishnan, 2015). IBD consists of two main diseases, ulcerative colitis (UC) and Crohn’s disease (CD), with approximately equal incidence (Ananthakrishnan, 2015). These two diseases can have very similar clinical presentations, with shared findings such as bloody diarrhea, abdominal pain and inflammation. However, despite these similarities, the two diseases respond to treatments differently. For example, although mesalazine is considered first-line treatment for UC to induce and maintain remission, its use in CD is controversial, with multiple studies failing to show efficacy (Akobeng and Gardener, 2005; Baumgart and Sandborn, 2007; Rasmussen *et al.*, 1987). These differences have prompted numerous attempts to understand the molecular characteristics and differences between CD and UC at the tissue level (Lawrance *et al.*, 2001; Wu *et al.*, 2007). An improved understanding of the molecular mechanisms of CD and UC has the potential to improve disease-specific treatment regimens, subtype patients for treatments, uncover new drug targets and increase the success of clinical trials.

Supervised machine learning has the potential to differentiate between UC and CD in active disease sites. Montero-Meléndez *et al.* (2013) (GSE36807) constructed a classifier using five genes (*FAM120A, GAS2L3, CPNE8, NQO2, HOXA10*), which yielded a 79% accuracy using leave-one-out cross-validation on colonic biopsies from 28 patients. However, this classifier has not been validated in any additional IBD datasets. There have also been studies that identify differentially expressed genes in UC and CD versus healthy controls, which could be used to construct gene signatures for classification (Dieckgraefe *et al.*, 2000; Lawrance *et al.*, 2001; Wu *et al.*, 2007). However, there is little concordance among the genes from these previous studies. Furthermore, when combining multiple studies, a meta-analysis concluded that inflammatory lesions in CD and UC are very similar, with essentially no gene differences found between the two diseases (van Beelen Granlund *et al.*, 2013). For differentiating similar polygenic diseases such as CD and UC, aggregating genes into a pathway-based approach has the potential to overcome these limitations.

Several methods implement pathway-based feature engineering approaches based on databases such as the Molecular Signatures Database (MSigDB) (Subramanian *et al.*, 2005) and the Kyoto Encyclopedia of Genes and Genomes (KEGG) (Kanehisa and Goto, 2000). Lee *et al.* (2008) identified specific differentiating genes within each pathway to aggregate into a pathway activity score. Su *et al.* (2009) calculated and aggregated the log-likelihood ratio (LLR) of genes within pathways. Recently, Young and Craft (2016) introduced a pathway-informed classification system and presented several methods of feature aggregation based on gene sets. However, these methods represent pathways as gene sets and ignore interactions between genes. Incorporating the underlying pathway architecture can improve performance and biological interpretability, particularly for distinguishing two diseases with many implicated genes in common. Indeed, methods that incorporate pathway topology for global pathway analysis, such as signaling pathway impact analysis, have shown superiority to traditional pathway over-representation analysis (Efroni *et al.*, 2007; Khatri *et al.*, 2012; Tarca *et al.*, 2009). These global pathway methods typically aggregate multiple samples per phenotype in order to identify significant biological pathways of interest and discover underlying biological mechanisms. The success of incorporating pathway topology in global pathway analysis has motivated the development of our method for individual pathway-based classification, which can be applied to a wide range of biological classification and prediction tasks.

In this work, we present a new, generalizable approach for individualized pathway-based classification, PRObablistic Pathway Score (PROPS), which uses Gaussian Bayesian networks to create individualized features that reflect pathway activity. We apply these pathway-based features to distinguish CD from UC. We compare our method against that of Montero-Meléndez *et al.*, as well as three additional gene-based approaches and four alternative pathway-based approaches. We demonstrate that our method produces superior performance in differentiating UC from CD and provides biological insight about the important pathways and the underlying molecular mechanisms driving these diseases.

## 2 Materials and methods

### 2.1 Datasets

We curated five datasets containing CD and UC patients at baseline. The first four, GSE6731 (Wu *et al.*, 2007), GSE9686 (Carey *et al.*, 2008), GSE10616 (Kugathasan *et al.*, 2008) and GSE36807 (Montero-Meléndez *et al.*, 2013), are publicly available studies downloaded from the Gene Expression Omnibus. An additional gene expression dataset of IBD patients from the Boston Children’s Hospital and the Brigham and Women’s Hospital (BCH/BWH) was provided by Pfizer Inc. (L.Afzelius, personal communication—manuscript in preparation). We selected these studies as they all contain both CD and UC tissue samples from areas of active disease. All studies were conducted with approval from their respective institutional review boards.

The BCH/BWH dataset was profiled using the Affymetrix PrimeView array. Multiple samples were taken from each patient from affected (lesional) and non-affected (non-lesional) areas of the small intestine and colon. For the 12 CD patients, there were a total of 42 non-lesional samples and 71 lesional samples. For the 13 UC patients, there were a total of 44 non-lesional samples and 75 lesional samples.

Data were normalized using robust multi-array average (Irizarry *et al.*, 2003), using the *affy* package (Gautier *et al.*, 2004) and R 3.2.3 (R Core Development Team, Vienna, Austria). We used ComBat (Johnson *et al.*, 2007) from the *sva* package (Leek *et al.*, 2016) to correct for batch effects across studies, where a batch consisted of an entire study (e.g. all samples from GSE10616), and phenotype labels were not used. We mapped probes to genes using the corresponding platform files, averaged genes that were associated with multiple probes and expanded probes that mapped to multiple genes so that the measured values for the probe contributed to the average value for each corresponding gene. Only genes measured across all studies contributed to the downstream analysis.

To be consistent across studies and limit effects due to anatomical location, we analyzed only colonic, non-rectal samples. GSE6731, GSE10616, GSE9686 and GSE36807 consist of only colon samples, and we excluded ileal and rectal samples from BCH/BWH, using the remaining 24 CD, 59 UC and 76 non-lesional samples. From GSE6731, we used the samples from affected areas from patients with a definitive diagnosis of CD or UC. All studies except for the BCH/BWH dataset contain healthy, UC and CD samples. The BCH/BWH dataset instead contains matched samples from non-lesional areas of the colon, which were used in addition to the healthy samples from other studies. BCH/BWH contains pediatric and adult samples, GSE9686 consists solely of pediatric samples and the remaining datasets are comprised of all adult samples.

### 2.2 PROPS feature engineering

We extracted all human pathways from KEGG, using the *KEGGgraph* package (Zhang and Wiemann, 2009). In KEGG, each pathway consists of a set of genes, which are represented by nodes and connected by directed edges. Missing genes that were not measured in all studies, and their corresponding edges, were not included. In order to convert the KEGG pathways into Bayesian networks, we started with the gene nodes and added the edges from KEGG in random order, excluding edges that would result in cycles. Only pathway networks with at least one edge were included. To quantify the fluctuations generated by this randomization, we tested 1000 additional random edge orderings per pathway. We calculated the index of dispersion of the generated values for each pathway over all IBD samples in the training dataset. We then constructed 1000 models from the training data and assessed the variance of the area under the curves (AUCs) in each validation dataset.

We modeled each KEGG pathway as a Gaussian Bayesian network, where a gene node represents the gene expression. We used the *bnlearn* package (Scutari, 2009) to model each node as a linear combination of its parent nodes, where all nodes are Gaussian. Let node X in the pathway G have parents $Y=\{Y_{1},Y_{2},\ldots ,\;Y_{n}\}$. Each node is modeled as follows:

$$P\left(X\;|\;|Y\right)∼N\left(\beta_{0}+\;\beta_{1}y_{1}+\beta_{2}y_{2}+\cdots +\beta_{n}y_{n};\sigma^{2}\right)$$

**Fig. 1.**
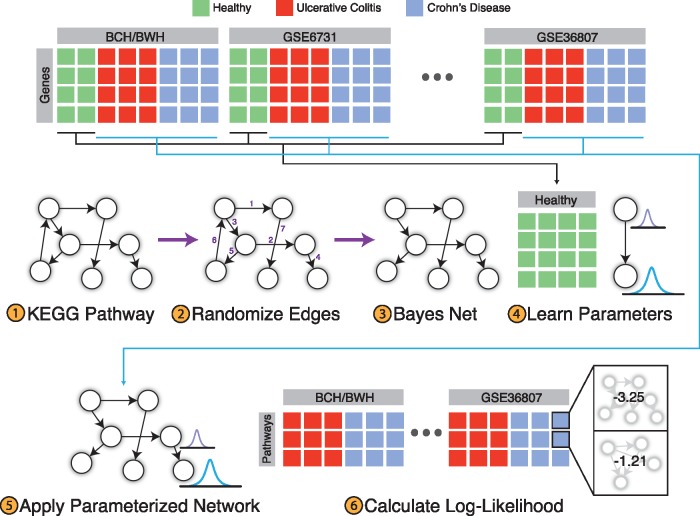
Overview of PROPS feature engineering. (**1**) KEGG pathways are downloaded and represented as directed networks. (**2**) Edges are added to the pathway in random order, excluding edges that would result in cycles. (**3**) This results in a Bayesian network representation of each KEGG pathway. (**4**) Each pathway model is parameterized using the healthy and non-lesional tissue samples. (**5**) The parameterized network is applied to CD and UC data to (**6**) calculate log-likelihood values for each pathway for each patient, which are used for subsequent classification

We then use the maximum likelihood estimate to learn the Gaussian distribution parameters and linear coefficients $\left\{\beta_{0},\beta_{1},\ldots ,\;\beta_{n},\;\sigma^{2}\right\}$ for each node using the healthy and non-lesional samples (Fig. 1). We apply the parameterized network model to the CD and UC samples, and for each pathway in each patient, we calculate the log-likelihood. Let $X=\{X_{1},X_{2},.,\;X_{n}\}$ be the nodes in pathway G. For a given sample for pathway G, let the data observation be $X_{1}=x_{1},X_{2}=x_{2},\ldots ,\;X_{n}=x_{n}$ and θ be the parameters of the pathway learned using the healthy and non-lesional data. Thus, the log-likelihood is calculated as follows:

$$\log\;P\left(X_{1}=x_{1},\ldots ,\;X_{n}=x_{n}\;|\;|\theta \right)=\sum_{i=1}^{n}\log\;P(X_{i}=x_{i}\;|\;\theta ,\;X_{pa}=x_{pa})$$

where $X_{pa}$ are the nodes in pathway G that are the parents of node $X_{i}$. These log-likelihood values are then used to represent each pathway as features for subsequent classification (Fig. 1).

### 2.3 Gene-based feature sets

For comparison, we implemented feature sets at the gene level, using the raw gene values. We created models using (i) all genes, (ii) all genes associated with a KEGG pathway (‘pathway genes’), (iii) the five genes from the classifier built by Montero-Meléndez *et al.* (‘GSE36807 five genes’) and (iv) the top 257 significant genes (‘top 257’). We chose 257 to match the number of features used in the pathway-based models. The GSE36807 five genes were present in all studies except in GSE6731 and were thus evaluated only in the remaining three studies, including GSE36807 itself.

### 2.4 Alternative pathway-based feature sets

For further comparison, we implemented four existing pathway-based feature engineering methods to compare against PROPS: LLR (Su *et al.*, 2009), conditionally responsive genes (CORG) (Lee *et al.*, 2008), gene expression deviation (GED) (Young and Craft, 2016) and normal tissue centroid (NTC) (Young and Craft, 2016). As detailed in the original papers, we used the canonical pathways in the C2 functional set of MSigDB (1329 pathways) for the former two and KEGG (257 pathways) for the latter two.

LLR calculates the ratio between the conditional probability density functions of CD samples versus UC samples for each gene, where both are assumed to be conditional Gaussian distributions. Next, the ratio is normalized across all samples. The pathway activity is then represented by the sum of normalized LLRs for all genes in the given pathway (Su *et al.*, 2009).

CORG identifies a set of genes that are most important for each pathway, and only those genes contribute to the pathway activity score. To determine which genes to include, CORG applies a greedy approach where genes are sorted by their *t*-test scores for CD versus UC, and genes are included sequentially until the discriminative score between CD and UC stops improving (Lee *et al.*, 2008).

NTC and GED are two approaches reported together that incorporate healthy samples when calculating pathway scores for additional phenotypes or conditions of interest (Young and Craft, 2016). For each sample, NTC represents each pathway as a location in gene space using the genes that are part of the pathway. The Euclidean distance between this point and the average healthy location is used as the measure of pathway activity. In contrast, GED creates two features for each pathway, one to represent the over-expressed genes and one for the under-expressed genes. A gene is included in the pathway score if the expression distribution for the phenotype of interest is significantly different from that of the healthy samples, using the Kolmogorov–Smirnov test. A score is calculated for those genes that are significantly differentially expressed, and this score is then added to either the over-expressed feature or the under-expressed feature based on its deviation from the healthy samples. The original implementation of NTC and GED has further parameters that exclude promiscuous genes, which are part of many pathways and limit pathways that are used in the final model via clustering silhouette scores (Young and Craft, 2016). We consider these to be tuning steps that are applicable to any of the methods described. To be consistent with all of the other methods that do not natively implement these tuning steps, we included all genes and pathways in our NTC and GED implementations.

### 2.5 Classifier construction and evaluation

For all nine feature sets, we constructed 100 random forest models using the *randomForest* package (Liaw and Weiner, 2002) and evaluated our classifiers using the median area under the receiver–operator characteristic (ROC) curve (AUC). We trained each model using the BCH/BWH dataset, which contains the most samples as well as both adult and pediatric samples, and used the four smaller publicly available datasets independently as external validation. We chose this setup over combining samples across studies in order to evaluate each dataset separately and limit batch effects when interpreting the results. We further compared performance between methods by aggregating the classification probabilities for all of the validation data, calculating the AUC and then using DeLong’s test (DeLong *et al.*, 1988) to assess significance. We only used independent validation data when assessing significance, and thus the GSE36807 five-gene classifier was assessed using only GSE10616 and GSE9686. Using these aggregate results, we further assess classification performance by constructing precision–recall curves and calculating the area under the precision–recall curve (AUPRC), where Crohn’s disease was designated as the positive class.

To assess feature importance, we extracted and averaged the mean decrease in the Gini index for each of our 100 models. We further evaluated our model by closely examining the incorrectly classified samples. We first visualized the classification results from PROPS using multidimensional scaling (Gower, 1966) with cosine distance. We then compared each pathway from the CD samples that were falsely classified as UC against the correctly classified CD samples using Student’s *t*-test. We did the same for the incorrectly classified UC samples.

## 3 Results

### 3.1 Gene expression and pathway data

We analyzed 7 UC, 5 CD and 4 healthy samples from GSE6731; 10 UC, 14 CD and 11 healthy samples from GSE10616; 5 UC, 11 CD and 8 healthy samples from GSE9686 and 15 UC, 13 CD and 7 healthy samples from GSE36807. From BCH/BWH, we used 24 CD and 59 UC samples, spanning 11 UC and 4 CD patients. We used all colonic, non-lesional samples from BCH/BWH, which consisted of 76 samples from 21 patients. Thus, for differentiating CD from UC, we trained on 83 samples with four validation sets containing 16, 24, 12 and 28 samples. There were 9116 genes common to all platforms used.

From KEGG, we extracted 300 human pathways. These pathways are composed of 7069 genes, of which 4561 were in our set of 9116. Only pathways with at least one edge were included, resulting in 257 pathways. For those methods that used MSigDB, we used all 1329 canonical pathways from the C2 functional set, version 5.2. These pathways contained 8899 genes, of which 5624 were in our set of 9116.

### 3.2 Probabilistic pathway score

**Fig. 2.**
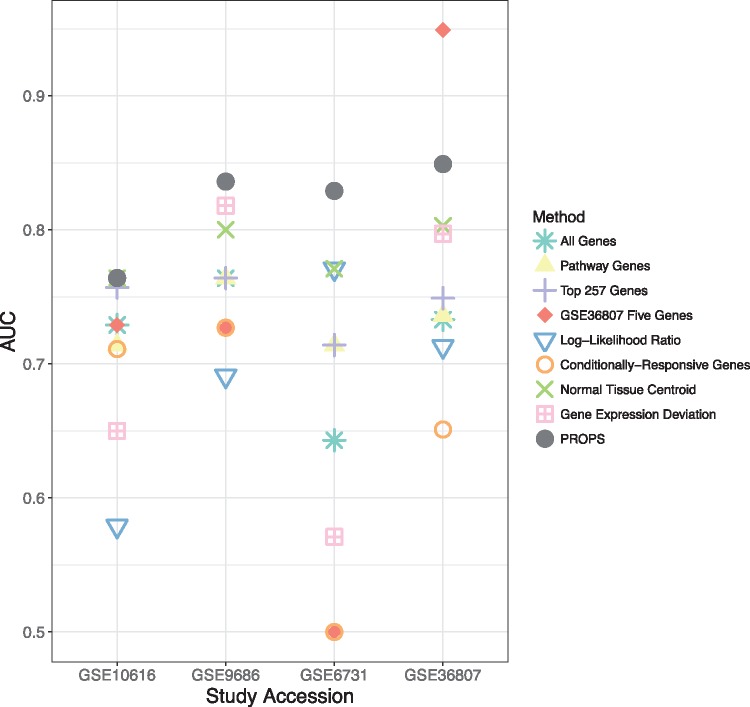
AUC comparison between all methods on all four validation datasets. PROPS consistently performs well and outperforms nearly all other methods in all studies

**Fig. 3.**
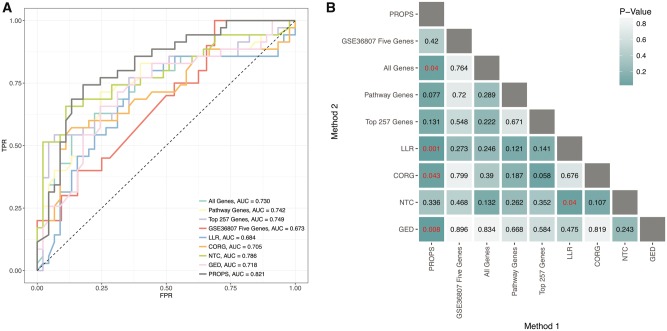
(**A**) Aggregate ROC curves and (**B**) pairwise AUC comparison between all methods on all independent validation data. For GSE36807 five genes, only GSE10616 and GSE9686 were used, resulting in fewer samples for comparison. PROPS obtains the highest AUC and outperforms more methods than all its competitors, significantly outperforming all genes, LLR, CORG and GED, and trending towards significance against pathway genes and top 257 genes

Using PROPS, we attained median AUCs of 0.764, 0.829, 0.836 and 0.849 in GSE10616, GSE6731, GSE9686 and GSE36807, respectively. Compared to the eight other methods, our method ranks first above all other methods in all the validation sets, with the exceptions of tying NTC in GSE10616 and being outperformed by the GSE36807 five genes in GSE36807. PROPS, on average, tended to outperform the gene-based feature sets by nearly 0.1 in the AUC (Fig. 2, Supplementary Fig. S1), NTC by 0.04 and other alternative pathway-based feature sets by 0.1 (Fig. 2, Supplementary Fig. S2). Aside from the two exceptions, PROPS outperforms all other models in all studies. When aggregating all the validation results, PROPS outperforms all other methods, with an AUC of 0.821 (Fig. 3A) and an AUPRC of 0.858 (Supplementary Fig. S3). PROPS statistically significantly outperforms LLR, CORG, GED and using all the genes (Fig. 3B). Though limited by the number of validation samples, particularly when comparing against the GSE36807 five genes, PROPS performs the best overall in pairwise comparison, surpassing at least four other methods, whereas the next best method, NTC, surpasses only LLR.

When converting the KEGG pathways to Bayesian networks, the mean proportion of edges kept was 95.5%. Our edge randomization sensitivity analysis over 1000 iterations yielded very small fluctuations in the log-likelihood values. For CD samples, the median index of dispersion was 0, with an interquartile range of 0–0.03. For UC patients, the median was 0, with an interquartile range of 0–0.003. After using the 1000 BCH/BWH sets to construct 1000 models, we found the variance in the AUC of the validation sets to be 0.00020 in GSE10616, 0.00016 in GSE6731, 0.00011 in GSE36807 and 0.00026 in GSE9686. We believe that fluctuations of this magnitude are unlikely to impact our model results or any other subsequent downstream analyses.

### 3.3 Gene-based features

The gene-based feature sets had median AUC on the validation sets ranging from 0.6 to 0.764, with the exception of the GSE36807 five genes with an AUC of 0.949 in GSE36807. However, it is clear that the model using the GSE36807 five genes trained by leave-one-out cross-validation in GSE36807 is overfit to that particular study, for it does not perform nearly as well on any of the other datasets. In fact, this model is generally surpassed by using all the genes or by expanding the number of top genes used (Fig. 2, Supplementary Fig. S1). Aside from this model, PROPS outperformed all gene-based feature sets across all four studies. Using the top 257 genes tends to produce the best performance out of all the gene-based feature sets, particularly in GSE10616 (Supplementary Fig. S1A). The improvement in the AUC was evident even when the sample size was small, for example in GSE6731 (Supplementary Fig. S1B).

### 3.4 Alternative pathway-based features

Our method outperforms the other alternative methods across all studies, with the exception of tying NTC in GSE10616 (Fig. 2, Supplementary Fig. S2). When comparing these methods across all of the validation data, our method performs significantly better than LLR, CORG and GED (Fig. 3B). Of the four alternative methods, NTC was the best performing pathway-based feature set across three out of the four validation sets, and GED obtained the best results in GSE9686 (Supplementary Fig. S2C). However, GED tended to be less consistent than NTC, as evidenced by GED underperforming against the gene-based methods in GSE6731 and GSE10616 (Supplementary Fig. S2A and B). LLR and CORG consistently performed below all the gene-based feature sets, except for LLR in GSE6731.

### 3.5 Model classification and important pathways

**Fig. 4.**
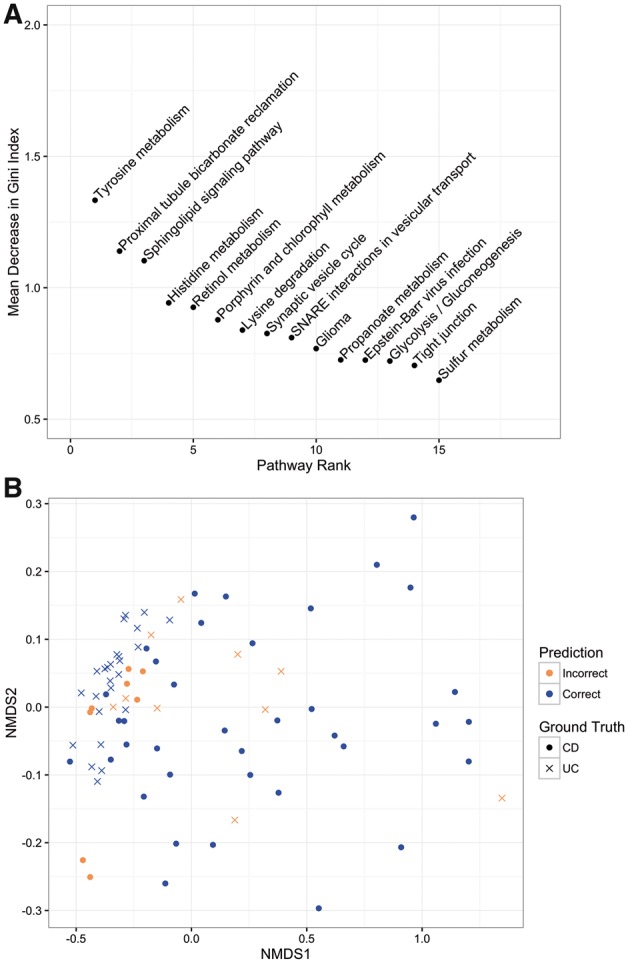
(**A**) The top 15 important features from our model. (**B**) Visualization of classification results using multidimensional scaling. The majority of the misclassified samples are located at the border between CD and UC, with five UC samples that appear to be more similar to CD than UC

The 15 most important pathways for classification as determined by our model are shown in Figure 4A. Interestingly, all of these pathways were found to be more perturbed (lower log-likelihood values) in UC as compared to in CD. The majority of pathways found are related to metabolism, many of which have been shown to play a role in IBD (Jansson *et al.*, 2009; Williams *et al.*, 2009).

We have visualized our PROPS features by projecting each sample into two dimensions and overlaying the classification results from our model (Fig. 4B). The CD samples misclassified as UC are generally located in the border region between the two diseases. However, there are a few UC samples (one from GSE9686, two from GSE10616 and two from GSE36807) that resemble CD samples more so than other UC samples. When comparing UC samples that were classified as CD to correctly classified UC samples, the five pathways that were most different were the chemokine signaling pathway; valine, leucine and isoleucine degradation; extracellular matrix receptor interaction; cytokine–cytokine receptor interaction and focal adhesion.

## 4 Discussion

In this work, we present PROPS as a novel method for creating individualized pathway scores based on a probabilistic framework. For each pathway, our method calculates the log-likelihood of each patient’s data, which we interpret as a measure of pathway perturbation and dysregulation. We apply our method to differentiate two similar, complex diseases, CD and UC, and show that our method achieves better performance than a previous CD versus UC classifier and multiple alternative gene and pathway-based methods. We use a random forest classifier, which was chosen to compare all nine methods as it can easily accommodate a wide range of number of features, can capture non-linear effects, requires less hyperparameter tuning than methods such as support vector machines and is relatively easy for users to implement and interpret. However, as PROPS is primarily a feature engineering method, any classification method may be used, which can be chosen to best suit the end user.

In contrast to previous individualized pathway-based methods, our method takes into account pathway topology, rather than treating pathways as gene sets. Additionally, our method does not prune genes or pathways based on user-defined thresholds or heuristics, making it easy to use with a consistent interpretation. There have been multiple global pathway analysis methods that incorporate pathway topology (Efroni *et al.*, 2007; Tarca *et al.*, 2009; Vaske *et al.*, 2010), where the latter two use underlying probabilistic graphs. However, both methods bin continuous data such as mRNA expression into discrete states, such as upregulated and downregulated. Determination of such thresholds to use for binning can be the cause of variance and subjectivity, and thus we have chosen instead to use continuous values in our method. However, use of continuous gene expression values without normalization or batch correction leads to incompatibility and bias when assessing experiments from different platforms and institutions. Thus, we perform batch effect normalization, where each study is one batch, as a pre-processing step, and have provided this functionality in our R package.

We use KEGG as our underlying pathway database, assuming that KEGG pathway topology is conserved across tissues and disease states, and that KEGG interactions are relevant at the mRNA expression level. We chose KEGG as it is frequently used to interpret gene expression data (Dahlquist *et al.*, 2002; Segal *et al.*, 2003) and for consistency as two of the competing methods also use KEGG. However, our method can use any set of directed pathways and can be used with different data sources, such as proteomics data. Graphical models have also been used to infer pathway structure from gene expression data (Dobra *et al.*, 2004; Massa *et al.*, 2010), and these resulting pathways are also compatible with our method. Using our data, we excluded some KEGG pathways because their genes were not measured in all five studies. Recent approaches in platform imputation may provide a solution for expanding the number of genes that can be used in downstream analysis (Zhou *et al.*, 2017).

In differentiating CD and UC at sites of active disease, we chose to use biopsy samples, as the number of studies containing resection samples is limited. Though CD is a transmural disease, mucosal healing has been correlated with improved outcomes and has been used as an endpoint in multiple clinical trials (Dave and Loftus, 2012; Rutgeerts *et al.*, 2007). Thus, understanding the differences in the mucosa, as captured by these biopsies, is important for distinguishing the disease mechanisms driving CD and UC.

In establishing the baseline pathway distributions, we used healthy controls and non-lesional samples. We included the non-lesional samples since van Beelen Granlund *et al.* (2013) showed that the profile of such samples was nearly identical to samples from healthy controls. Furthermore, we used samples from various locations, though all are from the colon. Previous studies specific to IBD have shown that there is no significant anatomical variation in the expression profiles among sites with active disease within the colon (Costello *et al.*, 2005; Wu *et al.*, 2007).

Overall, our method outperforms existing classifiers and methods, with an average AUC of 0.82 on four independent validation sets, an aggregate AUC of 0.821 and an AUPRC of 0.858. Our method surpassed the next best performing method by 0.035 in the aggregate AUC and 0.064 in AUPRC. Given that IBD is a relatively common chronic disease affecting an estimated 1.5 million Americans, a robust increase in performance, even if small, has the potential to affect a large number of patients. Notably, our method even performs well on small validation sets like GSE6731, which has only 12 samples. Additionally, our method is able to apply to pediatric (GSE9686) and adult samples (GSE6731, GSE10616 and GSE36807) and performs well even with different proportions of CD to UC patients in the validation set, as in GSE9686. Gene-based approaches, in contrast, tend to overfit, for example when using the five genes isolated from GSE36807 and when using all the genes. By using pathways, we are implementing biologically driven regularization in order to improve performance by condensing gene features into relevant, aggregate pathway features. These pathway-based features tend to be more robust and less prone to overfitting.

In addition to our novel methodology, our work also contributes biological insight into differentiating CD and UC. In distinguishing the two, we found that many of the top pathways are related to metabolism, as detailed below. Interestingly, pathways that are known to play a role in both diseases, such as immune-mediated and inflammatory pathways, do not appear to dominate this list. Such pathways likely have less discriminative power to separate these two diseases, since they are shared features. However, such pathways are important when examining UC samples that were misclassified as CD samples. These misclassified UC samples generally had higher log-likelihood values, differing on a few key IBD-related pathways from other UC samples. These samples may represent a difference in the disease state for these samples or a subtype of UC that more closely mimics CD at the molecular level.

Tyrosine metabolism is the top pathway with the highest mean decrease in the Gini index, surpassing the second most important pathway by a large margin. Nitration of tyrosine increases in the context of oxidative stress and inflammatory conditions (Hanazawa *et al.*, 2000; Kaur and Halliwel, 1994) and specifically in UC (Kimura *et al.*, 1998). Notably, Kruidenier *et al.* (2003) found a significant increase in immunohistochemical expression of 3-nitro-l-tyrosine in inflamed UC mucosa, but not in CD, non-inflamed UC or healthy controls. This is consistent with our finding of increased dysregulation of tyrosine in UC compared to CD. Nitrotyrosine has been implicated in damaging DNA and inflammation-mediated carcinogenesis (Murata and Kawanishi, 2004), which may be a contributing factor to the development of colorectal cancer, particularly in UC.

Aside from tyrosine metabolism, several of the other top pathways have a known role in IBD. For example, sphingolipids have been implicated in many inflammatory conditions (Maceyka and Spiegel, 2014), including IBD (Suh and Saba, 2015). Our results suggest that sphingolipid metabolism may play different roles in UC versus CD, with UC being more dysregulated than CD. One drug that targets this pathway, fingolimod, has been shown to prevent the development of colitis in mice (Deguchi *et al.*, 2006), and another drug, ozanimod, is currently being tested in clinical trials to treat UC. Many of the other top pathways have also been implicated in IBD, such as retinol metabolism (Reifen *et al.*, 2002), tight junctions (Edelblum and Turner, 2009) and sulfur and propanoate metabolism (Knights *et al.*, 2013). Although these pathways are known to have a role in IBD, our results suggest differing activity between CD and UC, and further investigation of the role of these pathways in the individual diseases is warranted.

## 5 Conclusions

In this work, we introduce a novel approach, PROPS, to calculate individual pathway-based scores, using Bayesian networks to capture pathway topology. We apply our method to differentiate CD and UC in order to elucidate disease mechanisms by harnessing information from sites of active inflammation. In distinguishing these two complex diseases, we demonstrate that PROPS is superior in performance and more robust than existing IBD classifiers and alternative methods, and that even pathways that are known to be shared by UC and CD show differing activity, which is useful for differentiation.

## Funding

This work was supported by the National Institutes of Health [R01 GM102365, T32 GM007365 and F30 AI124553] and Pfizer Inc. [IC2014-1387].

*Conflict of Interest*: none declared.

## Supplementary Material

Supplementary Data
