# Supplementary material for: A probabilistic pathway score (PROPS) for classification with applications to inflammatory bowel disease
Source: Bioinformatics. 2017 Oct 18;34(6):985–93. doi: 10.1093/bioinformatics/btx651 (PMC5860179; doi:10.1093/bioinformatics/btx651)
Supplement: Supplementary Data [file btx651_supplement_v1_proof.docx]

Supplementary Information

A PRObabilistic Pathway Score (PROPS) for Classification with Applications to Inflammatory Bowel Disease

Lichy Han^1^, Mateusz Maciejewski^2^, Christoph Brockel^3^, William Gordon^2^, Scott B. Snapper^4,5^, Joshua R. Korzenik^6^, Lovisa Afzelius^2^, Russ B. Altman^7,8,*^

^1^Biomedical Informatics Training Program, Stanford University, Stanford, CA 94305, ^2^Inflammation & Immunology, Pfizer Inc., 1 Portland Street, Cambridge, MA 02139, ^3^Hill’s Pet Nutrition, 1035 NE 43rd St, Topeka KS 66617, ^4^Division of Gastroenterology, Hepatology and Nutrition, Boston Children's Hospital, Harvard Medical School, Boston, MA 02115; ^5^Division of Gastroenterology, Brigham & Women's Hospital, Boston, MA, 02115, ^6^Department of Gastroenterology, Hepatology and Endoscopy, Brigham and Women's Hospital, Harvard Medical School, Boston, MA 02115, ^7^Department of Genetics and ^8^Department of Bioengineering, Stanford University, Stanford, CA 94305

*To whom correspondence should be addressed.

**
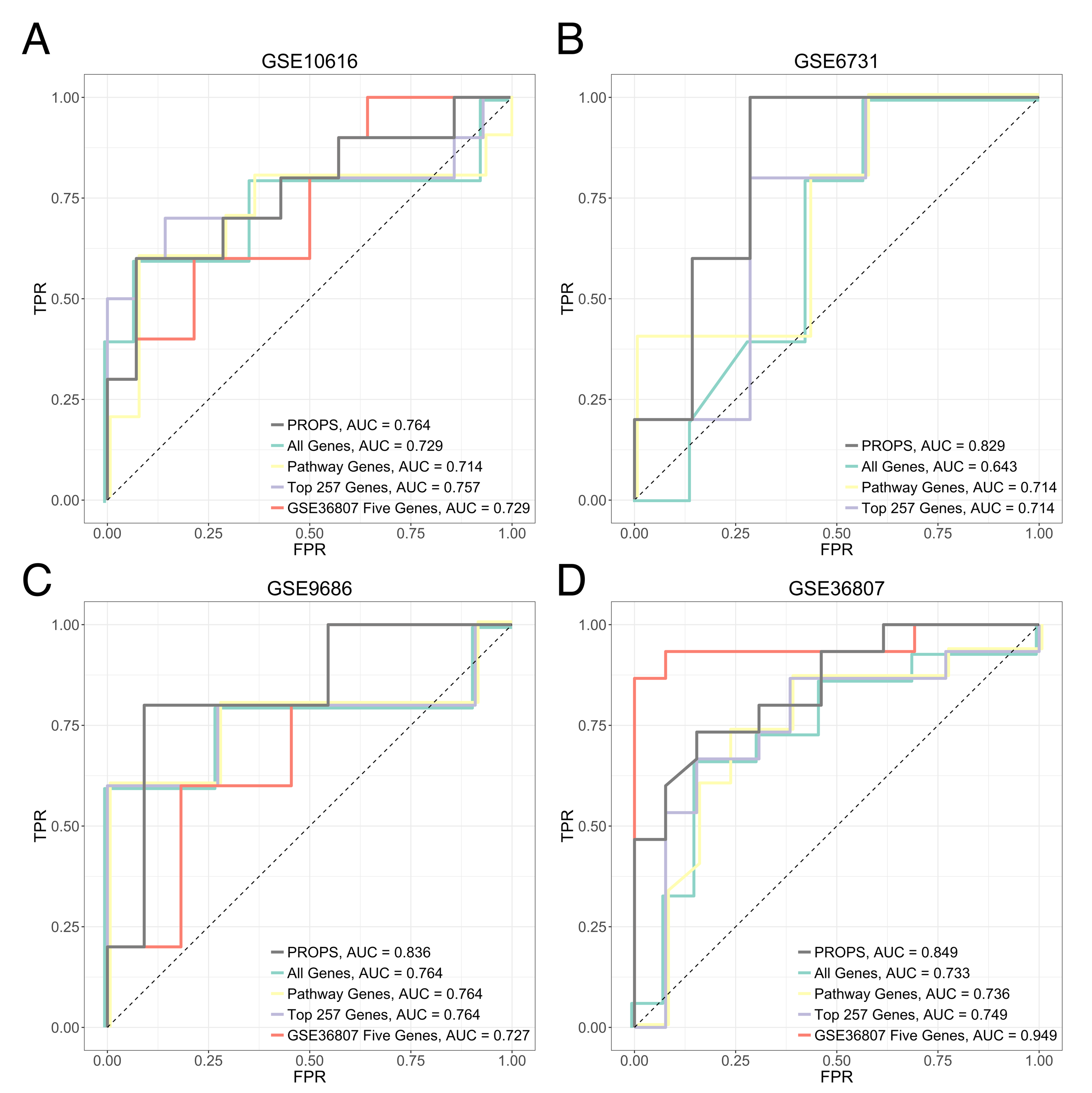
**

**Figure S1.** ROC curves for PROPS as compared to gene-based methods for GSE10616 (A), GSE6731 (B), GSE9686 (C), and GSE36807 (D). PROPS outperforms all gene-based methods in all studies, except for the GSE36807 five genes, which are overfit to GSE36807.


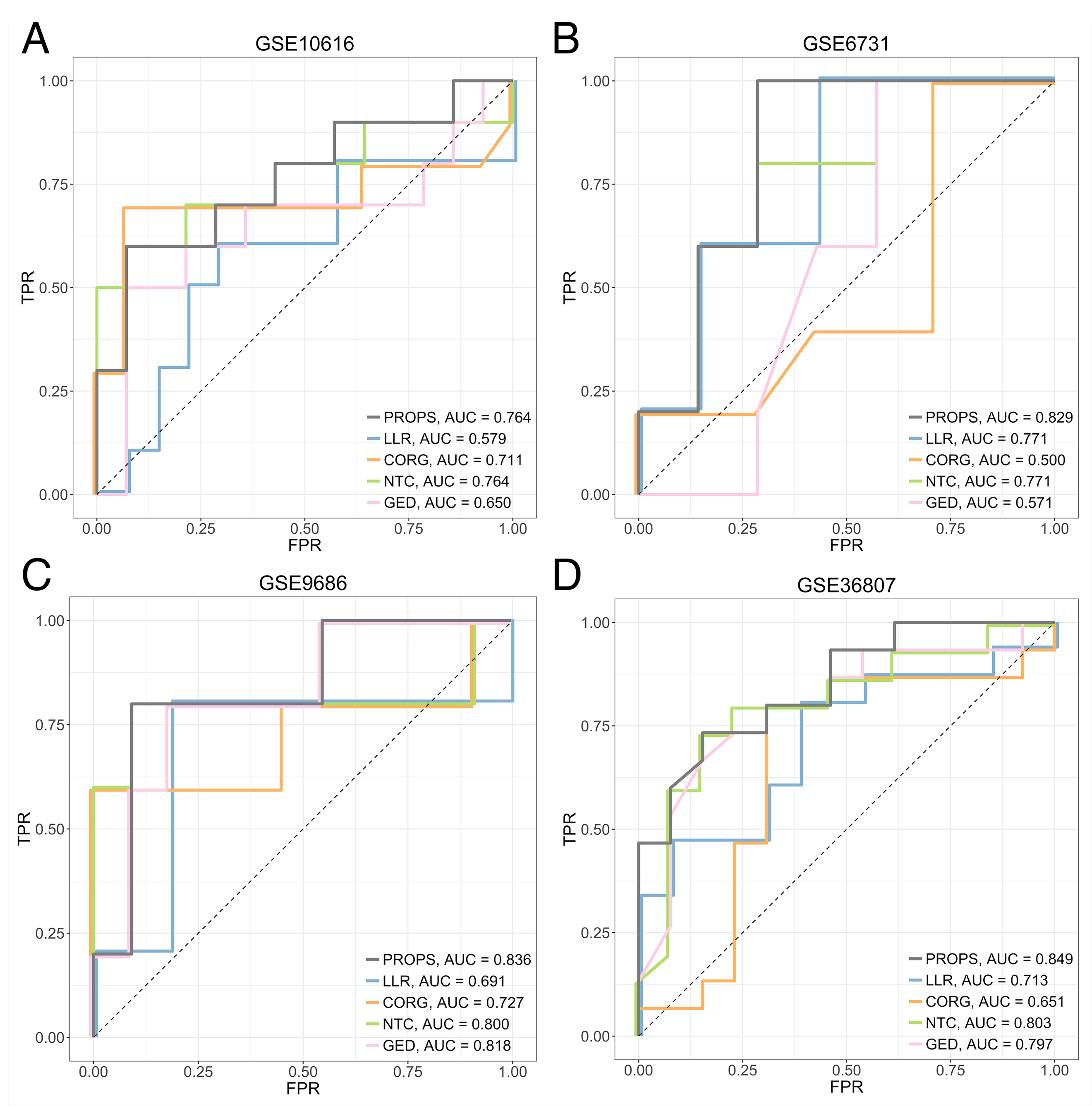


**Figure S2.** ROC curves for PROPS as compared to alternative pathway-based methods for GSE10616 (A), GSE6731 (B), GSE9686 (C), and GSE36807 (D). PROPS outperforms all alternative pathway-based methods in GSE9686, GSE36807, and GSE6731, and ties NTC in GSE10616.


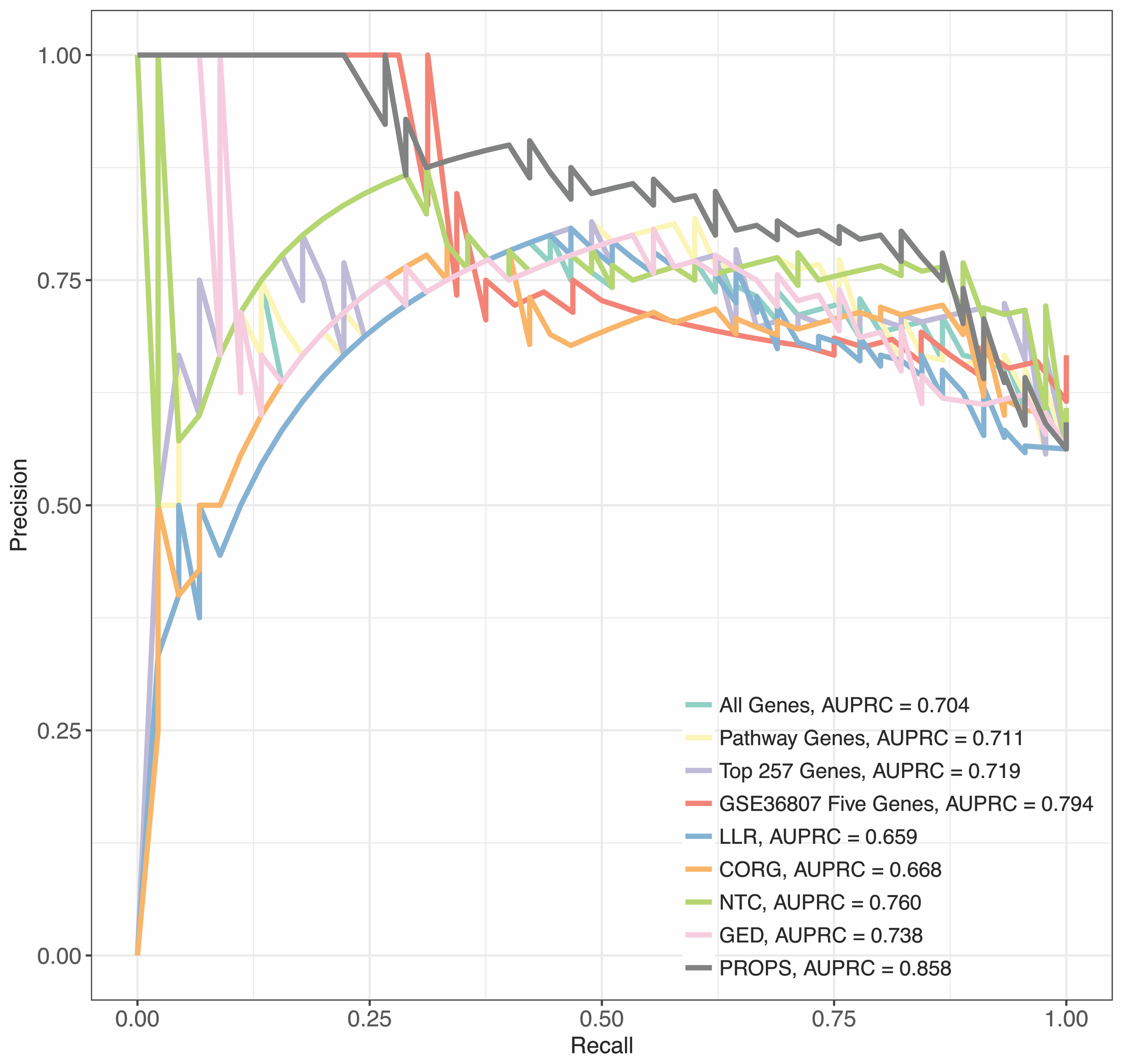


**Figure S3.** Precision-recall curves for all methods, where Crohn’s disease is considered the positive class and ulcerative colitis is considered the negative class. PROPS obtains the highest area under the precision-recall curve (AUPRC), surpassing all of the other eight methods.
